# Interfacial properties of a ZnO/PTFE composite from density functional tight-binding simulations†

**DOI:** 10.1039/d4ra06790h

**Published:** 2024-11-04

**Authors:** Chol Ryu, Jun-Gi Ri, Yun-Sim Kim, Chung-Hyok Rim, Chung-Il Kim, Chol-Jun Yu

**Affiliations:** a Chair of Computational Materials Design, Faculty of Materials Science, Kim Il Sung University Ryongnam-Dong, Taesong District Pyongyang Democratic People's Republic of Korea cj.yu@ryongnamsan.edu.kp

## Abstract

Metal-oxide-reinforced plastic nanocomposites are widely used in high-tech industries, but the reinforcement mechanism of the metal oxide is not fully understood. Here we investigate the interfacial properties of a zinc-oxide-reinforced amorphous polytetrafluoroethylene (a-PTFE) composite as a prototype for such composites using superlattice modeling and density functional tight-binding molecular dynamics simulations. To study the ZnO/a-PTFE composites, the superlattice supercells are built using a ZnO (112̄0) surface supercell and a-PTFE layer with an experimental density of 1.8 g cm^−3^ and various thicknesses. Our calculations demonstrate that the binding energy between ZnO and a-PTFE is negative, indicating their attractive binding, and electron accumulation occurs in the middle space between ZnO and a-PTFE, as well as around ZnO, evidencing that the newly formed interfacial chemical bonds are partially covalent. We further reveal that the tensile stress and elastic moduli of the ZnO/a-PTFE superlattice increases with increasing ZnO fraction, with values placed between those of ZnO and a-PTFE, which confirms the enhancement of the mechanical strength of the composites by incorporating ZnO into the a-PTFE matrix. This work provides a design guideline for developing high-performance metal-oxide-reinforced plastic composites.

## Abstract 2

We performed density functional tight-binding simulations of a ZnO/amorphous PTFE superlattice to reveal the interlayer binding strength, charge redistribution and enhancement of mechanical strength of the ZnO-reinforced PTFE plastic composites.

## Graphical abstract

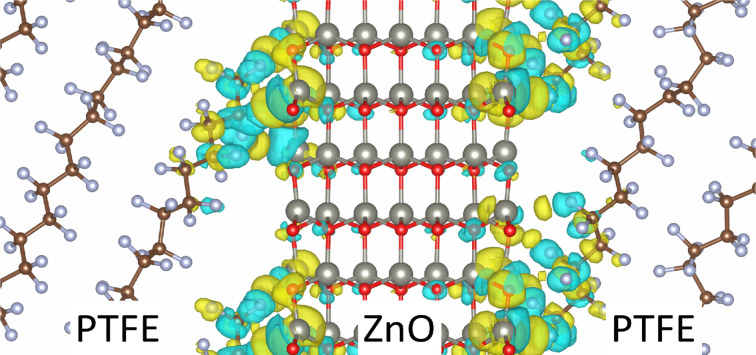

## 1 Introduction

Polytetrafluoroethylene (PTFE), known as “Teflon®” in commerce, has been attracting significant attention in various high-tech industries, such as chemical engineering, and the aerospace and automobile industries.^1^ This is due to the unique properties of PTFE, such as a low friction coefficient, excellent chemical resistance, high thermal stability and flame-retardant properties,^2^ which allow it to be used as a chemical catalyst,^3–5^ sealing agent,^6^ and surface coatings.^7–9^ In particular, the lowest friction coefficient of PTFE among solid polymers^10^ has made it the most promising substance for lubrication technology^11–15^ and triboelectric applications.^16–21^ However, pure PTFE has a low wear resistance and severe creep deformation,^22^ which limit its wide use in engineering. To address these problems, fibrous fillers, such as carbon fibers, glass fibers and whiskers,^23,24^ and spherical nanoparticles,^25^ have been incorporated into the PTFE matrix, leading to an increase in wear rate by one or two orders of magnitude.

Toward this aim, zinc oxide (ZnO) nanoparticles have been widely used as a reinforcing filler due to their excellent mechanical properties.^26^ In such polymer matrix composites, various polymers have been adopted as the matrix, such as PTFE,^18,27,28^ polyamides,^29^ polyurethanes,^30^ polyetheretherketone,^31^ polymethylmethacrylate,^32^ and ultrahigh molecular weight polyethylene (UHMWPE).^33^ Li *et al.*^28^ revealed that ZnO/PTFE composites with 15 vol% of ZnO show only 1% wear volume loss compared with pure PTFE. Chang *et al.*^33^ found that UHMWPE filled with 10 wt% ZnO nanoparticles has the optimal mechanical and tribological properties with remarkable enhancement in wear resistance and compressive strength compared with pure UHMWPE.

Materials modeling and simulations at the atomic scale can help to obtain understanding of a material’s properties and processes. Chen *et al.*^34^ performed molecular dynamics (MD) simulations to investigate the wetting behavior of water droplets on a PTFE surface. Using MD simulations, Barry *et al.*^35^ looked at the effect of temperature on the friction and wear of PTFE. In addition, first-principles calculations within the density functional theory (DFT) framework have been carried out to investigate the atomic structure and electronic properties of the crystalline PTFE compound^36^ and Al-PTFE contact electrification for a triboelectric nanogenerator.^20^ In spite of such computational studies for PTFE, theoretical studies for ZnO/PTFE composites are not yet found in the literature to the best of our knowledge. Therefore, the fundamental understanding of the interfacial properties of ZnO/PTFE composites remains unrevealed. The first-principles work for ZnO/PTFE composites will be of great importance in obtaining atomistic insights into tensile strength enhancement and chemical catalysis.

In this work, we report a first-principles study of an interface composed of ZnO bulk and amorphous PTFE (a-PTFE) using the density functional tight-binding (DFTB) method. Using the DFTB method and superlattice modeling,^37,38^ we investigate the interfacial bonding characteristics, interfacial energetics, and charge transfer at the ZnO/a-PTFE interface. The mechanical properties of the ZnO/a-PTFE composite are evaluated, including elastic constants and moduli, and the tensile strength is estimated when varying the ZnO fraction in the composite.

## 2 Methods

### 2.1 Superlattice modeling

**Fig. 1:**
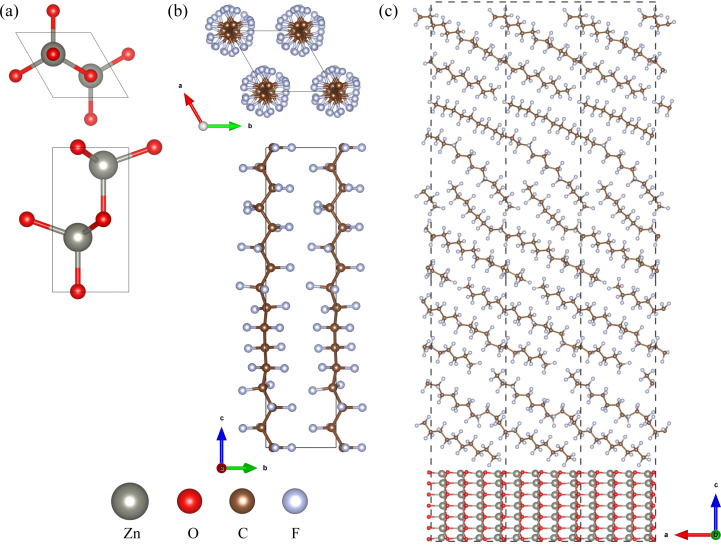
Ball-and-stick views of (a) a bulk ZnO unit cell in the hexagonal phase (space group *P*6_3_*mc*), (b) a c-PTFE unit cell in the hexagonal phase (space group *P*31), and (c) ZnO/a-PTFE supercells composed of a ZnO (112̄0) surface (2 × 1) supercell with 7 atomic layers and a-PTFE polymers filled in a 65 Å height space (33.67 wt% ZnO), where the dashed lines indicate the lattice.

To investigate the bulk properties of ZnO/a-PTFE composite, we adopted superlattice models, which consisted of a certain number of crystalline ZnO layers and an a-PTFE layer without a vacuum layer. For constructing the superlattice models, the interface was composed of a hexagonal ZnO surface and an a-PTFE polymer surface derived from its hexagonal crystalline phase (c-PTFE). Both ZnO and PTFE were crystallized in a hexagonal system with space groups of *P*6_3_*mc* and *P*31, respectively, as shown in Fig. 1(a) and (b). Among the different low index surfaces of ZnO, we selected the (112̄0) surface, which showed the lowest surface formation energy, as discussed in Section 3 (see Fig. S1, ESI†). For the ZnO/a-PTFE interface model, ZnO (112̄0) surface (2 × 1) supercells (size 11.508 × 5.430 Å) were constructed with 7 ZnO atomic layers and a vacuum layer with different thicknesses varying from 65 to 175 Å with an interval of 27.5 Å.

Then, the vacuum region of the supercell was filled with amorphous and randomly arranged PTFE polymers. To do this, a PTFE monomer with a length of 26 Å was firstly picked out from the crystalline phase and inserted in the vacuum region with an experimental a-PTFE density of 1.8 g cm^−3^,^39^ using the Amorphous Cell module of Materials Studio (version 8.0). While fixing the ZnO atomic layers and superlattice framework, the packing calculations including geometry optimizations were performed using the conjugate gradient method with the fine convergence tolerance and COMPASS (condensed-phase optimized molecular potentials for atomistic simulation studies) force field^40^ (see Fig. S2, ESI†). In these supercells of the ZnO/a-PTFE composites, the weight percentages of ZnO were determined to be 15.86, 18.03, 20.88, 25.78 and 33.67%, and these were named as model1 to model5, respectively. Fig. 1(c) shows the constructed supercell for the ZnO/a-PTFE model1 composite with 33.67 wt% ZnO among them.

### 2.2 Computational details

The DFTB calculations were performed using the DFTB+ package (version 21.2)^41^ and the 3ob-3-1 parameter set for the basic electronic Slater–Koster parameters.^42^ The DFTB method allows thousands of atoms to be treated at the quantum level, offering comprehensive and reliable prediction of complex material structures with manageable computational efficiency and reasonable accuracy. For Brillouin zone integration, the *k*-point meshes were set to 5 × 5 × 3 and 3 × 3 × 2 for the ZnO and c-PTFE bulk unit cells, respectively, and only Γ point was used for the ZnO/a-PTFE composite model. In order to take account of the van der Waals (vdW) interactions between adjacent PTFE molecules, the Tkatchenko–Scheffler dispersion model^43^ adapted for DFTB was used. In the geometry optimization, the atomic positions were relaxed until the force on each atom converged to 0.1 mHa bohr^−1^.

The tensile strength was determined by plotting a stress–strain curve, which was obtained by applying strain to the system along a certain direction, *i.e.*, elongating the supercell, and evaluating the resultant stress for each strain.^44^ To get the stress–strain information, DFTB-MD simulations were performed for the bulk ZnO supercell, and the a-PTFE and ZnO/a-PTFE superlattice supercells. After constructing the initial configurations, the initial supercell models were fully relaxed at room temperature of 298 K and an external pressure of 0 atm by performing NPT simulations for 3000 steps with a time step of 1 fs. Temperature and pressure in the NPT simulations were controlled by using a Nose–Hoover thermostat^45^ and Berendsen barostat,^46^ respectively. Then, NVT equilibrations were carried out at the same temperature for 3000 steps with a time step of 1 fs, using the Nose–Hoover thermostat. Finally, NVE simulations were carried out for 1000 steps with a time step of 1 fs. To speed up the MD simulations, we applied extended Lagrangian-type Born-Oppenheimer molecular dynamics (XLBOMD) as implemented in the DFTB+ package.^47^ In these XLBOMD simulations, the strain was gradually increased at a rate of 10^−5^ at intervals of 1 fs along the periodic direction. The mechanical properties such as bulk modulus *B*, shear modulus *G* and Young's modulus *E* were calculated using the ElaStic code^48^ in connection with the DFTB+ code.

For the surface-slab models, the surface formation energy was calculated using the following equation,1
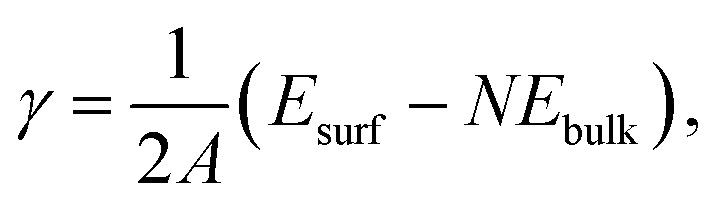
where *A* is the surface area, *E*_surf_ is the total energy of the surface slab, *E*_bulk_ is the total energy of the bulk unit cell per atom, and *N* is the number of atoms in the surface slab. To estimate the binding strength between ZnO and a-PTFE in the composite, the binding energy was calculated using the following equation,2
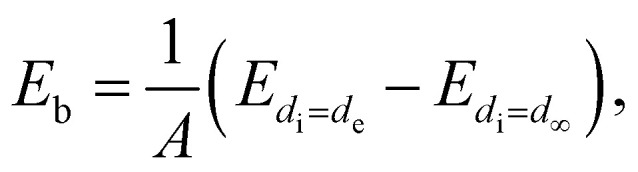
where *d*_i_, *d*_e_ and *d*_∞_ are the interlayer distance, equilibrium distance and infinity distance (∼20 Å), respectively.

## 3 Results and discussion

As a preliminary step, we performed structural optimizations for bulk ZnO and c-PTFE unit cells and compared the results with the experimental data, as shown in Fig. 1(a) and (b) (see Table S1, ESI†). For the ZnO unit cell, the optimized lattice constants with DFTB+ were *a* = 3.322 and *c* = 5.430 Å. Comparing with the experimental data of *a* = 3.250 and *c* = 5.207 Å,^49^ the relative errors (REs) are 2.2% and 4.3%, respectively. For reference, when compared with the results obtained by the pseudopotential plane-wave method as implemented in the Quantum ESPRESSO (QE) code (*a* = 3.284 and *c* = 5.302 Å), the DFTB+ calculations gave a slight overestimation. For the c-PTFE unit cell, the optimized lattice constants were *a* = 5.442 and *c* = 20.279 Å with RE values of −3.8% and 3.9% compared with the experimental data of 5.655 and 19.508 Å.^50^ Comparing with those obtained by the QE code (*a* = 5.763 and *c* = 19.761 Å), the DFTB+ calculations showed less accuracy but could be said to be reasonable with the maximum RE below 4.3%.

Then, the surface formation energies *γ* of the ZnO (0001), (101̄0) and (112̄0) surfaces were calculated using eqn (1) to select the plausible surface index and the number of atomic layers for the interface supercell. For the convergence test, *γ* was calculated as the number of atomic layers of surface slab supercells was increased (see Fig. S3, ESI†). It was found that the calculated *γ* values converged to 1.36, 0.87 and 0.89 J m^−2^ with 16, 8 and 4 atomic layers for the (0001), (101̄0) and (112̄0) surface slabs, respectively. When compared with those of the surface slabs with 28, 14 and 7 atomic layers, the absolute differences were found to be less than 0.01 J m^−2^. Therefore, the order of ZnO surface formability from the bulk is (0001) > (112̄0) ≈ (101̄0). Our calculations indicate that the non-polar ZnO (112̄0) surface has similar or lower surface energy than the polar (0001) and (101̄0) surfaces, which agreed well with the experimental fact that the (112̄0)-oriented ZnO surface is the most stable surface.^51^ Based on these findings, we chose the ZnO (112̄0) surface with 7 atomic layers to construct the supercells for the ZnO/PTFE superlattice models for the following further studies.

**Fig. 2:**
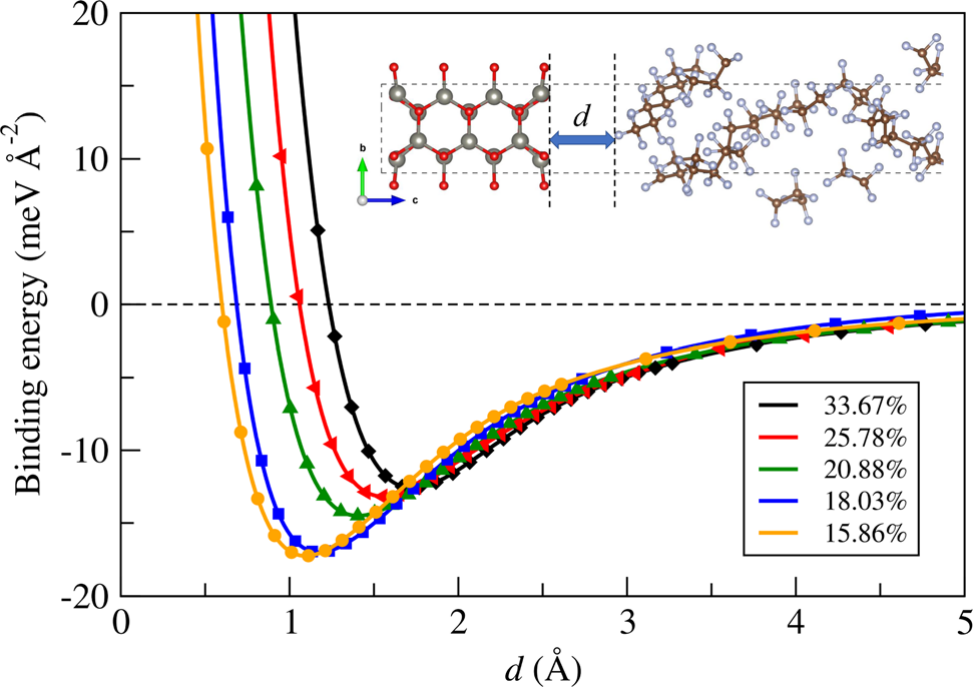
Binding energy between ZnO layer and a-PTFE layer as functions of interlayer distance *d* in the superlattice for ZnO/a-PTFE composites with different ZnO fractions.

Next, we calculated the binding energy between the ZnO layer and a-PTFE using eqn (2). Fig. 2 shows the binding energies as functions of interlayer distance in the superlattices for the ZnO/a-PTFE composites with different ZnO fractions, and Table 1 lists the determined values. For all the composite models, the binding energies were determined to be negative, indicating the attractive interaction between the ZnO layer and a-PTFE layer. Moreover, the short distance of *d*_ZnO–PTFE_ implies a strong interaction between the contacting layers due to a newly formed chemical bond at the interface. With increasing the PTFE content (or decreasing the ZnO weight percentage), the interlayer binding energy was found to gradually increase in magnitude while the interlayer distance decreased, indicating that the interaction between the ZnO and PTFE layers became stronger.

**Table 1:** Interlayer binding energy (*E*_b_) and distance (*d*), fracture stress and strain, and elastic moduli determined in the linear elastic region (0 ≤ *ε* ≤ 0.025) of the stress–strain curves for ZnO bulk, crystalline and amorphous PTFE, and ZnO/a-PTFE composites with the PTFE layer thickness of the supercell model, in comparison with the available previous works

Model	PTFE layer thickness (Å)	Weight fraction (%)		Interlayer binding		Fracture		Elastic modulus (GPa)	
		ZnO	PTFE	*E*_b_ (meV)	*d* (Å)	Stress (GPa)	Strain	This work	Prev. work
ZnO (*C*_11_)	—	100	0	—	—	25	0.15	193.62	209.7^a^, 206^b^
ZnO (*C*_33_)	—	100	0	—	—	25	0.23	203.16	210.9^a^, 209.5^b^
1	65.0	33.67	66.33	−12.59	1.73	9	0.22	80.14	
2	92.5	25.78	74.22	−13.18	1.56	8	0.20	76.35	
3	120.0	20.88	79.12	−14.50	1.42	6.5	0.20	61.06	
4	147.5	18.03	81.97	−17.01	1.25	5.8	0.25	39.98	
5	175.0	15.86	84.14	−17.26	1.10	4.5	0.20	45.59	
a-PTFE	—	0	100	—	—	4	0.23–0.34	30.30	1.6^c^
c-PTFE	—	0	100	—	—	13	0.19	130.41	221^d^, 247^e^

a Ref. 52.

b Ref. 53.

c Ref. 54.

d Ref. 55.

e Ref. 56.

**Fig. 3:**
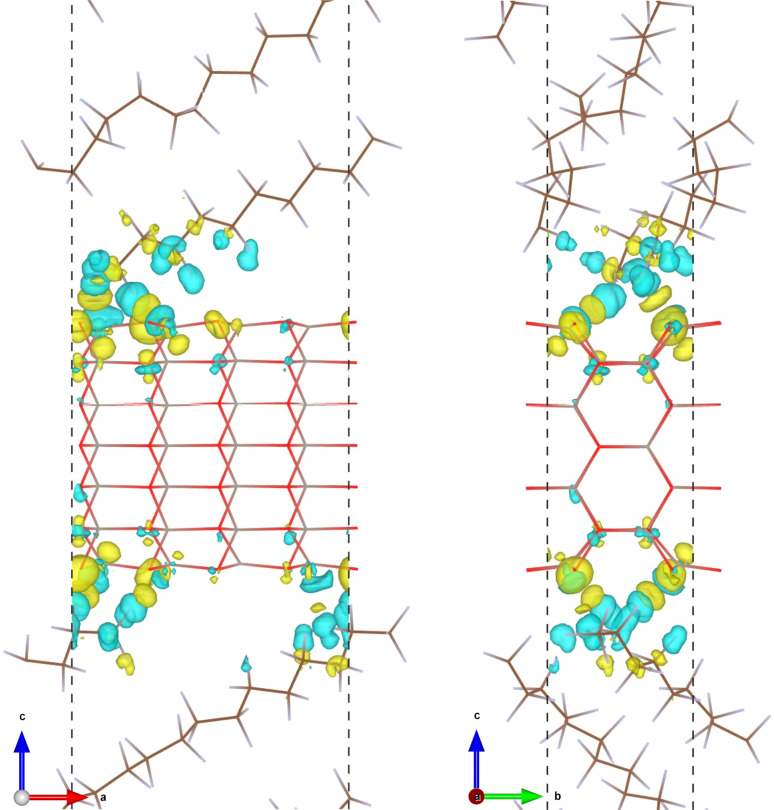
Isosurface view of electron density difference at the value of 0.001|*e*| Å^−3^ upon the interface formation of ZnO/a-PTFE model1 with a ZnO fraction of 33.67 wt%. The yellow (cyan) color represents the charge accumulation (depletion).

The formation of an interfacial chemical bond is accompanied by charge transfer and electron density redistribution, which can be confirmed by plotting the electron density difference. The electron density difference was calculated by using the following formula, Δ*ρ*(**r**) = *ρ*_int_(**r**) − [*ρ*_ZnO_(**r**) + *ρ*_PTFE_(**r**)], where *ρ*_int_(**r**), *ρ*_ZnO_(**r**) and *ρ*_PTFE_(**r**) are the electron densities of the ZnO/a-PTFE interface, ZnO, and PTFE surfaces, respectively. Fig. 3 shows the isosurface view of the electron density difference at the value of 0.001|*e*| Å^−3^ for the case of ZnO/a-PTFE superlattice with a ZnO fraction of 33.67 wt%, where the yellow (cyan) color represents the electron accumulation (depletion). The charge accumulation was observed around the oxygen atoms of the ZnO utmost layer, while charge depletion was found around the fluorine atoms of the a-PTFE chains toward the ZnO surface, indicating that some electrons were transferred from a-PTFE to the ZnO layer. Also, a remarkable amount of charge accumulation was found in the middle space between the ZnO layer and a-PTFE layer, implying that the newly formed interfacial chemical bond is covalent.

**Fig. 4:**
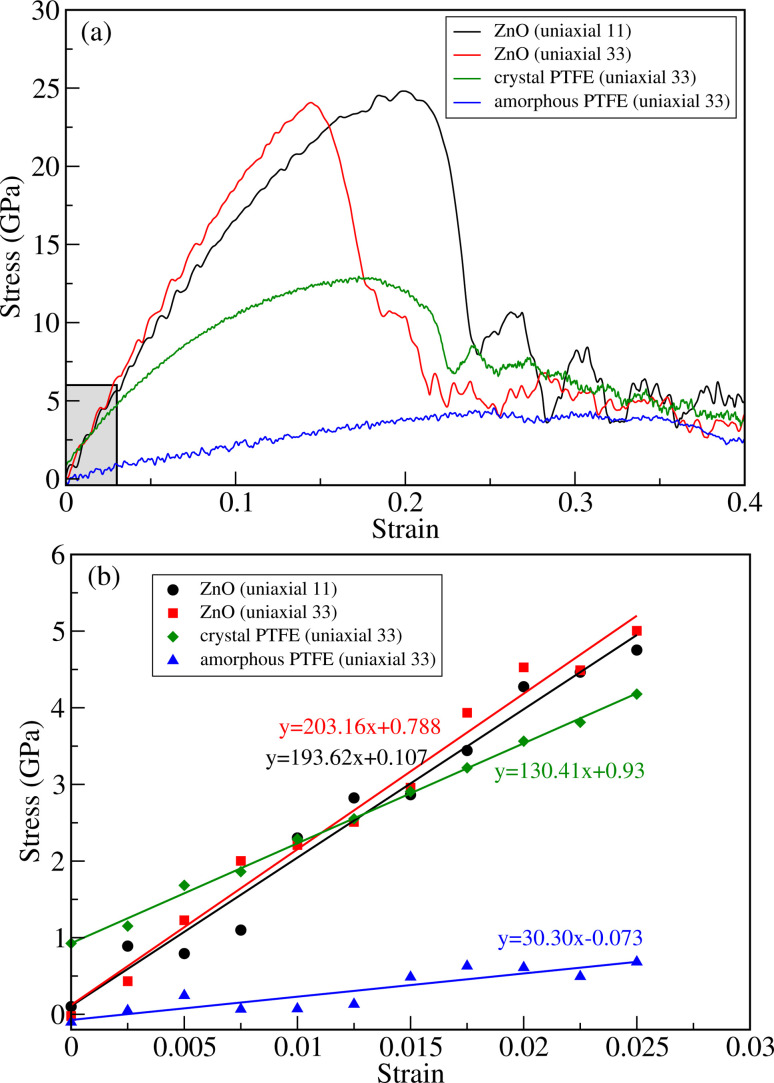
(a) Stress *versus* strain curves for bulk ZnO subject to axial (11) and transverse (33) loads and for crystalline and amorphous PTFE subject to axial load (33). (b) Linear stress *versus* strain relation in the linear elastic stage below 0.025 as shown in the shaded region in (a).

To evaluate the tensile strength, we obtained stress *versus* strain curves by performing DFTB-MD simulations at room temperature of 298 K. Firstly, we performed the simulations for bulk ZnO and crystalline and amorphous PTFE for comparison with the ZnO/a-PTFE composites. Fig. 4 show the obtained stress–strain graphs for ZnO subject to the uniaxial loads along the (11) and (33) directions and for PTFE along the (33) direction. As can be seen from Fig. 4, all the stress–strain curves have a linear region indicating that the compound is in a linear elastic stage. After the linear region, the stress was found to oscillate and fluctuate with increasing strain, indicating that the material had entered the plastic deformation stage. Finally, when the stress value reached a maximum, a sharp decline of stress followed, at which point the material was fractured (see Fig. S4 for equilibrated and fractured structures, ESI†). The maximum stress and the corresponding strain were defined as fracture stress *σ*_f_ and fracture strain *ε*_f_, respectively.

Table 1 lists the determined fracture stress and strain values. From the obtained stress–strain curves for bulk ZnO, the fracture stress was found to be about 25 GPa under both the axial (11) and transverse (33) strains. Although the fracture stress was the same, the fracture strain values were found to be quite different: *ε*_f_ = 0.15 and 0.23 for the transverse and axial strains, respectively. This indicates the anisotropy in the mechanical properties of the crystalline ZnO bulk. Meanwhile, the fracture stress was found to be about 13 GPa at the fracture strain value of about 0.19 for c-PTFE. The higher value of fracture stress in ZnO than that in a-PTFE or c-PTFE indicates that bulk ZnO is mechanically stronger than PTFE and thus can be used as a reinforcement for PTFE matrix-based composites. For the case of a-PTFE, the maximum stress was obtained as 4.2 GPa at the relatively wide range of strain between 0.23 and 0.34, although the sharp decline of stress was not observed. At the values of strain beyond 0.34, an onset of necking leading to fracture was observed.

The elastic moduli of ZnO and PTFE were calculated by applying the least-squares method within the linear elastic region, where the strain varied from 0 to 0.025, as shown Fig. 4(b). For the case of ZnO bulk, the linear interpolation gave the equations of *y* = 193.62*x* + 0.107 (GPa) and *y* = 203.16*x* + 0.788 (GPa) for the axial (*C*_11_) and transverse (*C*_33_) strains, respectively. For the case of PTFE, the linear equations were found to be *y* = 130.41*x* + 0.93 (GPa) and *y* = 30.30*x* − 0.073 (GPa) for the crystalline and amorphous phases, respectively. From these obtained linear equations, the elastic moduli were determined to be 193.62 and 203.16 GPa for bulk ZnO under the axial and transverse strains, and 130.41 and 30.30 GPa for the crystalline and amorphous PTFE phases, respectively. The elastic constants and moduli were also calculated by using the ElaStic code in connection with DFTB+ code and QE code, confirming reasonable agreement between them (see Tables S2 and S3, ESI†).

For the case of bulk ZnO, the elastic moduli of 193.6 and 203.2 GPa obtained from the linear fitting of the stress–strain curves in the linear elastic stage were close to not only those of 181.8 and 202.8 GPa calculated using the ElaStic code in connection with DFTB+ and QE codes but also those of 209.7, 210.9,^52^ 206 and 209.5 GPa (ref. 53) in previous works. For the case of c-PTFE, the elastic modulus of 130.4 GPa calculated using linear fitting was slightly underestimated compared with those of 188.5 and 190.3 GPa obtained using the ElaStic code in connection with DFTB+ and QE codes and those of 221 (ref. 55) and 247 GPa.^56^ For the case of a-PTFE, the linear fitting gave an elastic modulus of 30.3 GPa in agreement with the value of 22.3 GPa obtained using the ElaStic plus DFTB+ code. However, the calculated values were much larger than the experimental value of 1.6 GPa,^54^ which might be due to the adopted computational parameters and limited simulation cell size of a-PTFE. Nevertheless, we emphasize that the elastic modulus of a-PTFE is remarkably smaller than that of c-PTFE, indicating that our computational setting can be acceptable for estimating the tensile strength of ZnO/a-PTFE composites.

**Fig. 5:**
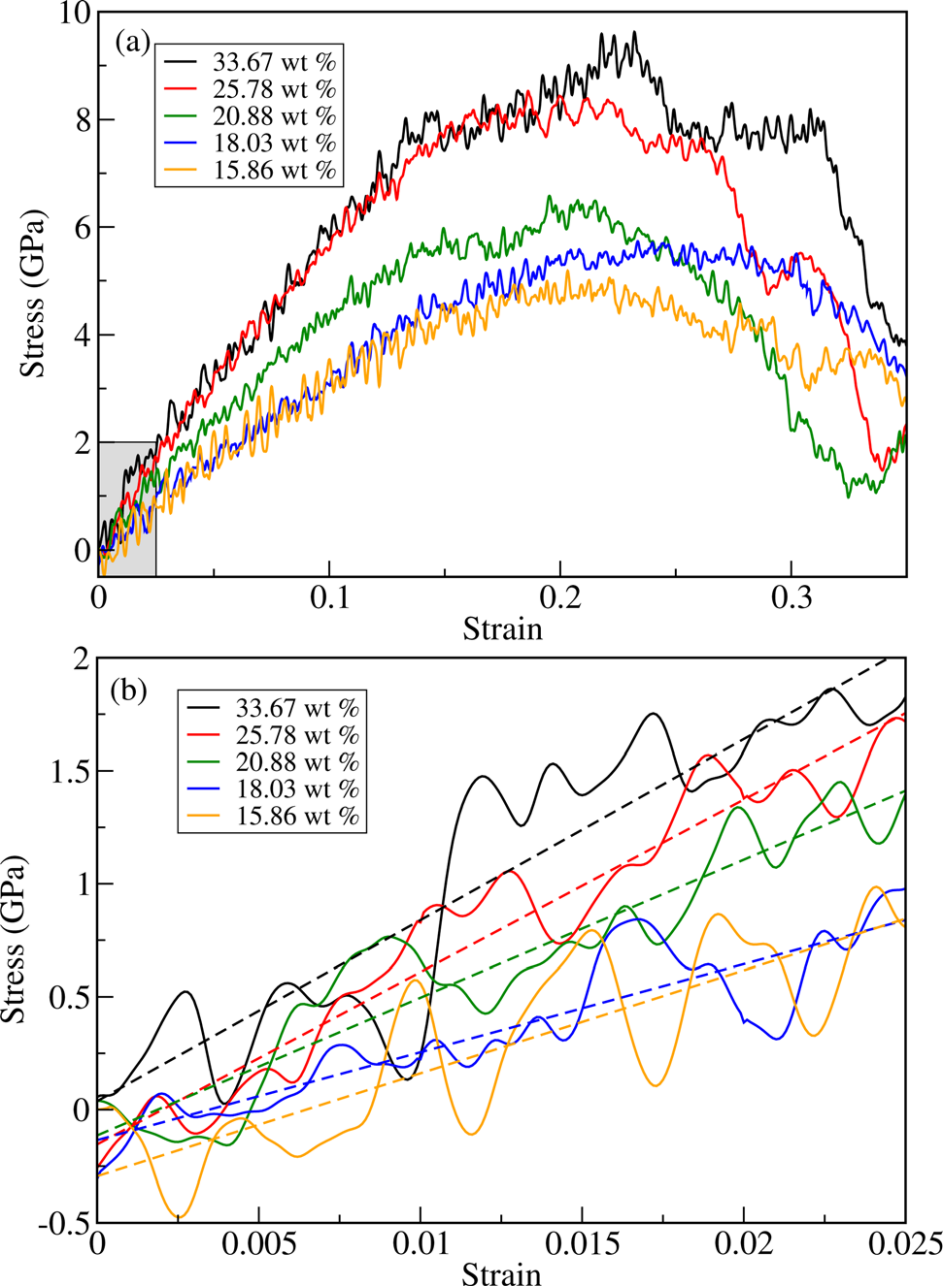
(a) Stress *versus* strain curves for ZnO/a-PTFE composites with different ZnO fractions subject to axial load. (b) Linear stress *versus* strain relation within a low-strain region below 0.025, as shown in the shaded region in (a). Dashed straight lines indicate linear fitting results.

As can be seen in Fig. 4 and Table 1, the fracture stress and elastic modulus of ZnO bulk are significantly larger than those of a-PTFE, verifying that ZnO used as a reinforcing agent is mechanically much stronger than a-PTFE used as a matrix. Therefore, when mixing ZnO materials into a PTFE matrix to make ZnO/PTFE composites, the mechanical strength can be expected to be between that of ZnO and a-PTFE, and enhanced compared with a-PTFE. Fig. 5(a) shows the stress–strain curves obtained by performing DFTB-MD simulations for ZnO/a-PTFE composite models with different ZnO fractions. The linear fitting of the curves within the low strain region below 0.025 was also performed to determine their elastic moduli, as shown in Fig. 5(b). As listed in Table 1, the Young's moduli of the ZnO/a-PTFE composites gradually increased from 39.98 GPa for model4 (ZnO fraction of 18.03%) to 80.14 GPa for model1 (ZnO fraction of 33.67%) upon increasing the ZnO fraction. We note that the order of model4 and model5 was changed, which might be due to the numerical noise of the DFTB-MD simulations Such increasing tendencies were also observed in the bulk and shear moduli (see Table S4, ESI†). The maximum tensile stresses of the ZnO/a-PTFE composite were also found to increase from 4.5 GPa for model5 to 9 GPa for model1 upon increasing the ZnO weight percentage in the composite, as listed in Table 1. However, the fracture strain was found to show an irregular trend, having values of 0.20, 0.22 and 0.25, which are similar to those in ZnO and c-PTFE but lower than that in a-PTFE. We note that the determined fracture stresses of ZnO/a-PTFE superlattices were smaller than that of ZnO bulk but larger than that of a-PTFE, indicating the enhancement of mechanical strength by introducing ZnO into the a-PTFE matrix.

## 4 Conclusions

In this work, we have investigated the interfacial properties of ZnO/a-PTFE composites while systematically varying the ZnO fraction by using superlattice models and DFTB calculations, aiming at clarifying the reinforcing role of a ZnO agent in the a-PTFE matrix composites. The supercells for the ZnO/a-PTFE superlattices were constructed using ZnO (112̄0) surface (2 × 1) supercells with 7 atomic layers and a-PTFE with a density of 1.8 g cm^−3^ and different thicknesses from 65 to 175 Å. Our calculations demonstrated that binding between ZnO and a-PTFE was attractive due to the negative binding energy and that covalent interfacial chemical bonds were newly formed upon the interface formation by the transfer of electrons from a-PTFE to ZnO while accumulating charge in the middle space between ZnO and a-PTFE. Furthermore, we revealed that the mechanical strength properties such as tensile stress and elastic moduli of the ZnO/a-PTFE composites could be clearly enhanced by incorporating ZnO with higher values as reinforcement into a-PTFE with lower values as the matrix by obtaining stress–strain curves through DFTB-MD simulations. With these findings, this work contributes to our understanding of the reinforcement mechanism of ZnO in a-PTFE-based composites and gives a design guideline for developing high-performance metal-oxide-reinforced plastic composites.

## Author contributions

Chol Ryu performed calculations and post-processing, and drafted the first manuscript. Jun-Gi Ri, Yun-Sim Kim, Chung-Hyok Rim and Chung-Il Kim assisted with the DFT calculations and contributed to useful discussions. Chol-Jun Yu developed the original project and supervised the work. All authors reviewed the manuscript.

## Conflicts of interest

There are no conflicts to declare.

## Supplementary Material

RA-014-D4RA06790H-s001
