# Supplementary material for: Interfacial properties of a ZnO/PTFE composite from density functional tight-binding simulations
Source: RSC Adv. 2024 Nov 4;14(47):35097–103. doi: 10.1039/d4ra06790h (PMC11533984; doi:10.1039/d4ra06790h)
Supplement: RA-014-D4RA06790H-s001 [file RA-014-D4RA06790H-s001.pdf]

Supplementary Information (SI) for RSC Advances.
This journal is © The Royal Society of Chemistry 2024

Electronic Supplementary Information – Interfacial properties of ZnO/PTFE composite from density functional tight binding simulations†

Chol Ryu, Jun-Gi Ri, Yun Sim Kim, Chung-Hyok Rim, Chung-Il Kim and Chol-Jun Yu*

Chair of Computational Materials Design, Faculty of Materials Science, Kim Il Sung University, Ryongnam-Dong, Taesong District, Pyongyang, Democratic People's Republic of Korea

Table S1. Lattice constants of ZnO unit cell in hexagonal phase with space group of $P63mc$ and crystalline PTFE (c-PTFE) unit cell in hexagonal phase with space group of $p31$, calculated with Quantum Espresso (QE) and DFTB+ codes, in comparison with the experimental data. The relative error (RE) is provided.

Compound	Method	$a = b$ (Å)	RE (%)	c (Å)	RE (%)
ZnO	DFTB+	3.322	2.220	5.430	4.290
	QE	3.284	1.051	5.302	1.832
	Exp.[a]	3.250		5.207	
c-PTFE	DFTB+	5.442	−3.767	20.279	3.952
	QE	5.763	1.910	19.761	1.297
	Exp.[b]	5.655		19.508	

[a]S. C. Abrahams and J. L. Bernstein, Acta Crystallogr. B 25, 1233–1236, 1969.
[b]V. M. Bouznik, *et al.*, Powder Diffr. 19, 219–224, 2004.

Table S2. Elastic constants of bulk ZnO and crystalline PTFE (c-PTFE) calculated by using the ElaStic code in connection with DFTB+ code and QE code (unit: GPa).

Compound	Method	C_{11}	C_{33}	C_{44}
ZnO	DFTB+	181.8	211.3	33.7
	QE	202.8	219.8	38.9
c-PTFE	DFTB+	11.1	188.5	1.5
	QE	16.3	190.3	18.1

Table S3. Elastic moduli of ZnO bulk, crystalline and amorphous PTFE determined with linear fitting of stress–strain curves in the linear elastic stage in comparison with those determined by using the ElaStic code in connection with DFTB+ code and QE code (unit: GPa).

	Linear fitting	ElaStic with DFTB+	ElaStic with QE
ZnO (C_{11})	193.62	181.8	202.8
ZnO (C_{33})	203.16	211.3	219.8
c-PTFE (C_{33})	130.41	188.5	190.3
a-PTFE (C_{33})	30.30	22.3	–

Table S4. Young's modulus (E), bulk modulus (B) and shear modulus (G) calculated using the ElaStic code in connection with DFTB+ for the ZnO/a-PTFE composites with gradually increasing the ZnO fraction (unit: GPa).

ZnO fraction (wt.%)	E	B	G
0	22.30	11.56	9.46
15.86	39.74	18.43	17.42
18.03	44.22	19.22	19.80
20.88	46.41	21.53	20.34
25.78	51.32	24.16	22.39
33.67	62.69	30.45	27.09
100	181.79	124.95	72.28

*cj.yu@ryongnamsan.edu.kp

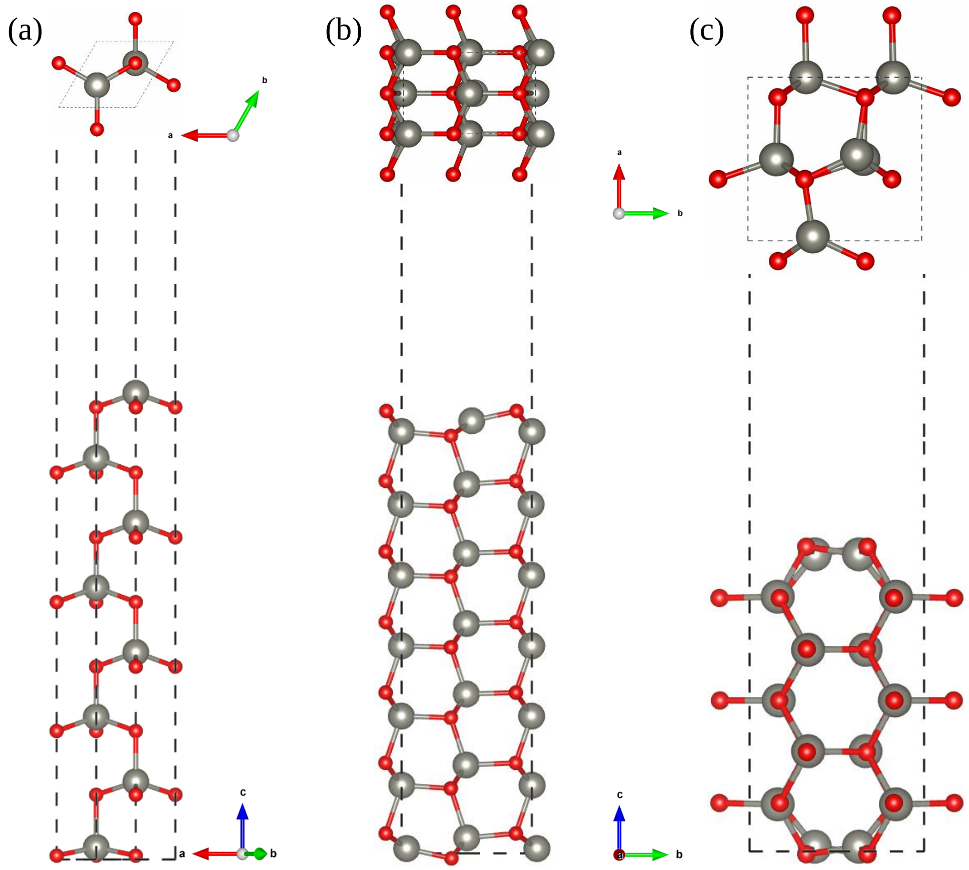

Figure S1. Optimized structures of ZnO surface slab supercells with top and side views for (a) (0001), (b) $(10\bar{1}0)$ and (c) $(11\bar{2}0)$ surfaces.

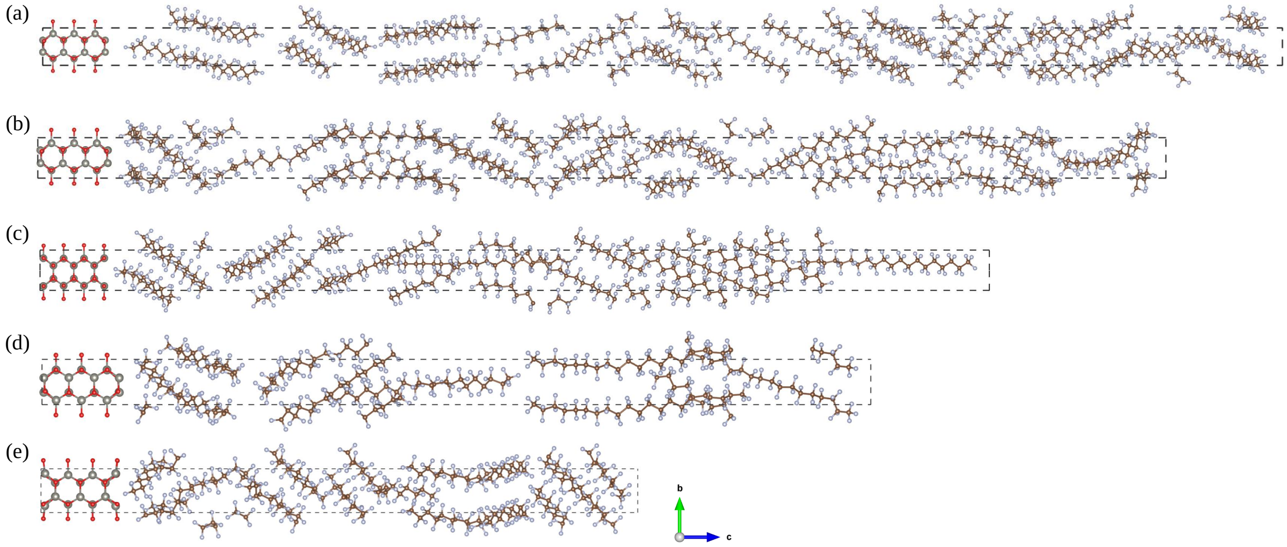

Figure S2. Optimized structures of ZnO/PTFE composites with different ZnO weight percent: (a) 15.86%, (b) 18.03%, (c) 20.88%, (d) 25.78%, (e) 33.67%.

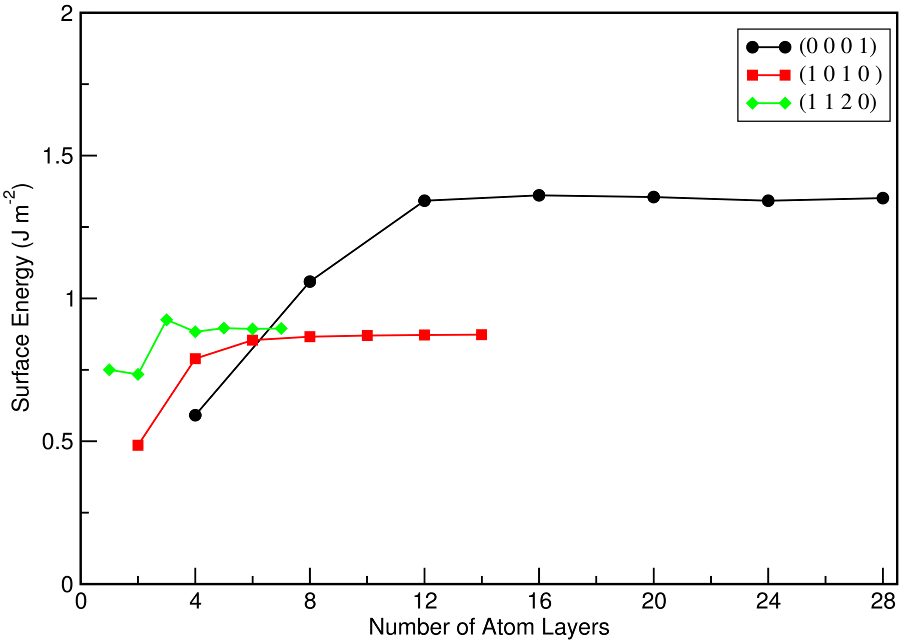

Figure S3. Convergence test for surface energies of ZnO (0001), (10$\bar{1}$0) and (11$\bar{2}$0) surfaces as increasing the number of atomic layers, calculated with DFTB+.

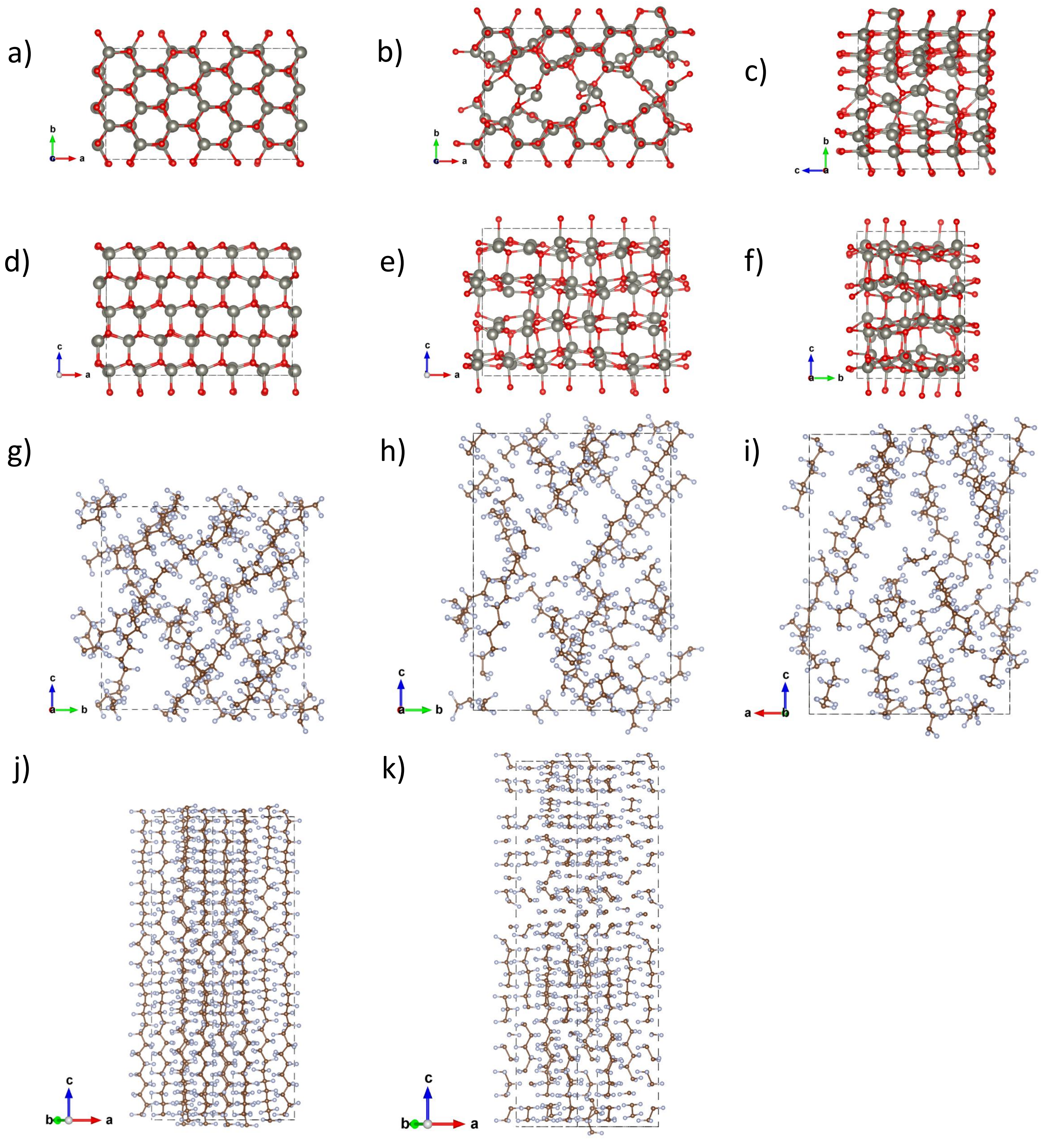

Figure S4. Ball-and-stick view for the equilibrated and fractured structures. (a) Equilibrated ZnO structure on *a*-*b* plane and (b, c) fractured ZnO structure under the strain load along the [010] direction. (d) Equilibrated ZnO structure on *a*-*c* plane and (e, f) fractured ZnO structure under the strain load along the [001] direction. (g) Equilibrated *a*-PTFE structure on *b*-*c* plane and (h, i) fractured *a*-PTFE structure under the strain load along the [010] direction. (j) Equilibrated *c*-PTFE structure and (k) fractured *c*-PTFE structure under the strain load along the [010] direction.
